# Energy Metabolism and Metformin: Effects on Ischemia-Reperfusion Injury in Kidney Transplantation

**DOI:** 10.3390/biomedicines12071534

**Published:** 2024-07-10

**Authors:** Denise V. Nemeth, Leonardo Iannelli, Elena Gangitano, Vito D’Andrea, Maria Irene Bellini

**Affiliations:** 1 School of Osteopathic Medicine, University of the Incarnate Word, San Antonio, TX 78235, USA; 2 Department of Surgery, Sapienza University of Rome, 00161 Rome, Italy; 3 Department of Experimental Medicine, Sapienza University of Rome, 00161 Rome, Italy

**Keywords:** metformin, ischemia-reperfusion injury, kidney transplant

## Abstract

Metformin (MTF) is the only biguanide included in the World Health Organization’s list of essential medicines; representing a widespread drug in the management of diabetes mellitus. With its accessibility and affordability being one of its biggest assets, it has become the target of interest for many trying to find alternative treatments for varied pathologies. Over time, an increasing body of evidence has shown additional roles of MTF, with unexpected interactions of benefit in other diseases. Metformin (MTF) holds significant promise in mitigating ischemia-reperfusion injury (IRI), particularly in the realm of organ transplantation. As acceptance criteria for organ transplants expand, IRI during the preservation phase remain a major concern within the transplant community, prompting a keen interest in MTF’s effects. Emerging evidence suggests that administering MTF during reperfusion may activate the reperfusion injury salvage kinase (RISK) pathway. This pathway is pivotal in alleviating IRI in transplant recipients, potentially leading to improved outcomes such as reduced rates of organ rejection. This review aims to contextualize MTF historically, explore its current uses, pharmacokinetics, and pharmacodynamics, and link these aspects to the pathophysiology of IRI to illuminate its potential future role in transplantation. A comprehensive survey of the current literature highlights MTF’s potential to recondition and protect against IRI by attenuating free radical damage, activating AMP-activated protein kinase to preserve cellular energy and promote repair, as well as directly reducing inflammation and enhancing microcirculation.

## 1. Introduction

For more than half a century, metformin (MTF, 1,1-dimethylbiguanide hydrochloride) has been, as a monotherapy or in combination with other drugs, a first-line regimen for type 2 diabetes mellitus (T2DM) [1]. MTF represents the only biguanide included in the World Health Organization (WHO)’s list of essential medicines [2], due to its efficacy and high safety profile. Many health economic studies have evaluated and confirmed its cost-effectiveness, making it a valuable tool in the management of diabetes mellitus [3]. Over time, an increasing body of evidence has emerged regarding additional roles of MTF, with benefits, not yet fully understood, in many pathologies including obesity, hepatopathologies [4], cardiovascular diseases [5], rheumatoid arthritis [6], different types of cancer [7] (e.g., breast and colorectal), polycystic ovarian syndrome [8], and viral diseases such as COVID-19 [9]. Additionally, MTF has been shown to be an anti-aging drug [10], and last but not least, a potential therapy for ischemia-reperfusion injury (IRI), an inevitable consequence of the transplantation process.

## 2. History and Uses of Metformin

MTF traces its roots back to the plant *Galega officinalis*, also known as “goat’s rue” or “French lilac” [11]. *G. officinalis* is part of the *Fabaceae* family of perennial herbs, which ranks as the second most diverse among plant families globally, with its species spanning across various regions. These plants boast a wealth of phytochemicals such as flavonoids, lectins, saponins, alkaloids, carotenoids, and phenolic acids and thus offer numerous health advantages [12]. *Galega officinalis* is considered to be native to Europe and southwest Asia and since medieval times has been known mainly for its hypoglycemic properties [13]. These properties are attributable to the presence of guanidine which is a nitrogen-rich chemical compound that occurs naturally in mollusks, marine sponges, earthworms, rice hulls, and turnip juice [14].

**Figure 1:**
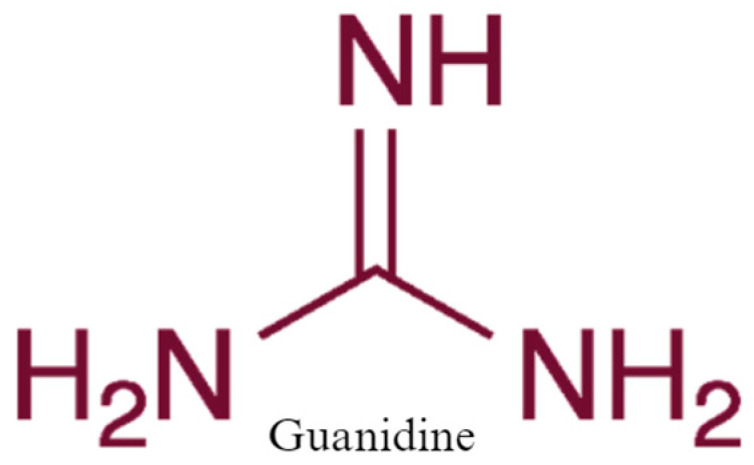
The chemical structure of guanidine.

Unlike carbonic acid, guanidine is fairly stable under normal conditions and does not necessarily require being in solution or cryogenic temperatures to obtain this stability. It is important to note that guanidine is distinct from guanine (Figure 1), a purine derivative present in bat and bird feces [15]. Guanidine is widely recognized as one of the most potent organic bases, with a pKa value of 13.6 [16].

It was discovered in late 1800 by Adolph Strecker, a German chemist best known for his valuable work specific to amino acids. Other discoveries attributable to him are those of compounds such as choline and the first successful synthesis of an amino acid in a laboratory, a fortunate blunder while he attempted to synthesize lactic acid [17]. The latter is best known as the “Strecker Synthesis” which yielded alanine as the final product of his reaction.

Guanidine-containing organic compounds are widespread in nature, appearing in various forms such as the neurotransmitter agmatine, alkaloids such as tetrodotoxin, and the amino acid arginine [18]. Due to their ability to form noncovalent interactions with molecular agents and proteins, coupled with their adaptability to their environment allowing them to vary in pKa value, they are viewed as a versatile group of compounds. As mentioned previously, the guanidinium group is highly stable, a characteristic that can be largely attributed to what is known as Y-aromaticity [19,20]. The hypoglycemic action of guanidine was demonstrated in animal studies in 1918 [21]. Numerous drugs containing guanide are effective in lowering blood sugar levels, yet many of them pose a significant risk of toxicity. As a result, only metformin, among the guanide-based medications, has maintained a widespread and long-term use in modern clinical settings [22].

**Figure 2:**
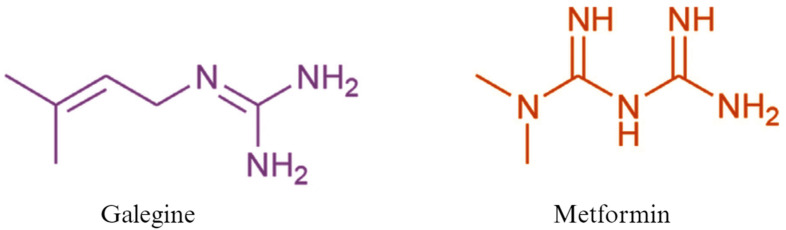
The chemical structures of galegine and metformin.

When guanidine proved excessively toxic, research shifted focus to galegine (Figure 2), an isoprepenyl derivative of guanidine, which underwent human trials in the 1920s, as a potential hypoglycemic treatment. Additional studies have revealed that galegine also possesses hypotensive effects, antifeedant properties, and body weight regulation effects [23,24]. The hypotensive effects of galegine are thought to be tied to its agonist effect on H2-receptors, which are intracellular G-protein coupled receptors typically associated with gastric secretion. However, through research, it has been discovered that numerous extra gastric H2-receptors can be found in the cardiovascular system, gastrointestinal muscles, endocrine and exocrine glands, brain, and pulmonary systems, amongst many other unexpected sites [25]. The agonism of H2-receptors will result in a heightened adenylate cyclase system activity which will cause a subsequent rise in intracellular cyclic AMP levels. At the vascular level, this leads to vasodilation causing the hypotensive effects previously discussed [26]. Despite the current data available, additional research must be conducted including tests on drug receptor-specific interaction and chronic toxicity assessments in order to validate the long-term safety of galegine as an antihypertensive therapy [24]. In light of the previous research and after guanide was abandoned due to toxicity, two new galegine-derived compounds, metformin and phenformin (biguanides), were synthesized [27]. Following studies assessing their hypoglycemic potential in animal models and finding that any significant glucose-lowering effects necessitated exceptionally high doses of the compound, these two were subsequently overlooked for over a decade [28]. In the 1940s, guanidine-containing agents regained attention due to their potential antimalarial effects, primarily with the antimalarial agent proguanil. Proguanil, a guanidine-containing antimalarial agent, also demonstrated glucose-lowering effects in animal trials. It should be noted that metformin’s antimalarial properties continue to be studied to date. In 2019, Vera et al. demonstrated that MTF treatment decreases *Plasmodium falciparum* growth in human hepatocytes and concluded that combining metformin treatment with suboptimal doses of conventional antimalarials proves more effective in reducing parasite load [29]. Furthermore, Eusebio Garcia found metformin to be effective in treating a local influenza outbreak in the Philippines. This led to the emergence of metformin hydrochloride’s application as an anti-influenza agent, referred to as Flumamine [30]. In the process, metformin’s tendency to reduce blood sugar levels in some patients was observed [8]. This marked a significant turning point in the narrative surrounding metformin. However, a puzzling aspect of this claim is that no blood glucose levels were actually documented for Garcia’s patients and there was a lack of experimental evidence supporting his theory on the blood glucose-lowering effects of metformin. Despite this, metformin’s fame endured, eventually leading French physician–scientist Jeane Sterne, who had been trained in diabetology in Paris, to undertake further studies. Sterne’s work in 1957 was the key to establishing metformin as a hypoglycemic medication definitively, marking a pivotal moment in metformin’s clinical history [28]. His publication acknowledged the oral hypoglycemic metformin in humans. Nevertheless, metformin continued to garner little attention due to its lower potency compared to the biguanides of phenformin and buformin. These two compounds were phased out in the late 1970’s due to the heightened risk of lactic acidosis. As metformin faced an uncertain future, its association with these discontinued medications tainted its reputation. Eventually, its ability to combat insulin resistance and address adult-onset hyperglycemia without causing weight gain or increasing the risk of hypoglycemic events gained recognition in Europe. Following Sterne’s report, metformin was introduced in the United Kingdom in 1958 as a treatment for TDM2 under the trade name Glucaphage^®^, meaning ‘glucose eater’. However, its approval by the Food and Drug Administration (FDA) for use in the USA came much later in the mid 1990’s [31,32]. Again, this delay likely attributed to the withdrawal of phenformin with a high incidence of lactic acidosis and cardiovascular side effects. Today, metformin is considered the ‘gold standard’ and the preferred drug for managing patients with T2DM.

Additionally, MTF has been suggested as an adjunct in the treatment of malignancy, as research has shown that it has the potential to counteract the “Warburg Effect” at the cellular level, a phenomenon in which cancer cells choose to utilize the glycolytic pathway, converting glucose into lactate to meet their energy requirements, irrespective of oxygen availability [33]. Metformin accomplishes this via the inhibition of the mTORC1 pathway, which supports cancer, through both AMPK-dependent and independent mechanisms [34], thus inhibiting tumor growth.

Further research has demonstrated that metformin has anticancer effects both in vivo and in vitro [35]. Evidence from in vivo studies has surfaced, demonstrating an antiproliferative effect on breast cancer and there is some evidence to suggest that this effect might be independent of its metabolic effects, directly inhibiting tumor growth [36]. Studies suggest that the most significant advantages are observed in colorectal and prostate cancer patients, especially those undergoing radical radiotherapy. However, randomized controlled trials examining metformin dose, duration, and efficacy are strongly recommended.

## 3. Pharmacokinetics of Metformin

MTF is the most common orally administered drug for the treatment of T2DM [37]. Its prescription rate is known to be as high as 45–50% of all prescriptions. Currently, more than 150 million people take metformin [38]. In the US, the amount of metformin prescriptions doubled in 2021, accounting for 90 million prescriptions [39]. This trend can largely be attributed to the significant rise in diabetes cases worldwide. In 2021, approximately 537 million people were living with diabetes globally, and projections suggest that by 2045, this number will exceed 783 million [40].

MTF is available both as an immediate-release formulation, which is usually administered twice daily (bid), and as extended-release formulations, which are administered once (qd) or twice daily [41]. While both formulations have similar efficacy and significant differences in side effects [42], some studies have shown that immediate-release formulations have a superior glycated hemoglobin (HbA1c)-lowering capability, in addition to improving triglyceride levels and total cholesterol levels [43]. Considering that gastrointestinal (GI) side effects are the primary cause of non-adherence to and the discontinuation of MTF treatment, extended-release formulations offer significantly improved GI tolerability [44]. Other less commonly seen adverse effects of metformin include flushing, palpitations, headaches, drug eruptions, and vitamin B12 deficiency, amongst others [45].

Initial dosages of immediate-release MTF are 500 mg qd or bid with meals and should be increased gradually according to tolerance until a target dose of 1000 mg bid is reached [41]. Metformin is contraindicated in patients with an eGFR below 30 mL/min/1.73 m^2^ [1]. Following oral intake, MTF is predominantly absorbed in the small bowel, with a bioavailability (F) of 55–60% [46]. It is worth emphasizing that because the liver receives blood directly from the portal vein, it can accumulate a significantly higher concentration of orally administered metformin compared to the bloodstream and other organs [47]. After absorption, it is distributed to tissues, such as the liver and kidneys, where it is eliminated through both filtration and tubular secretion without undergoing modification (half-life = 4–5 h) [46]. Its peak plasma concentration happens about 2–3 h after a 500 mg dose of immediate-release formulation and 6–8 h of extended-release formulation [48].

**Figure 3:**
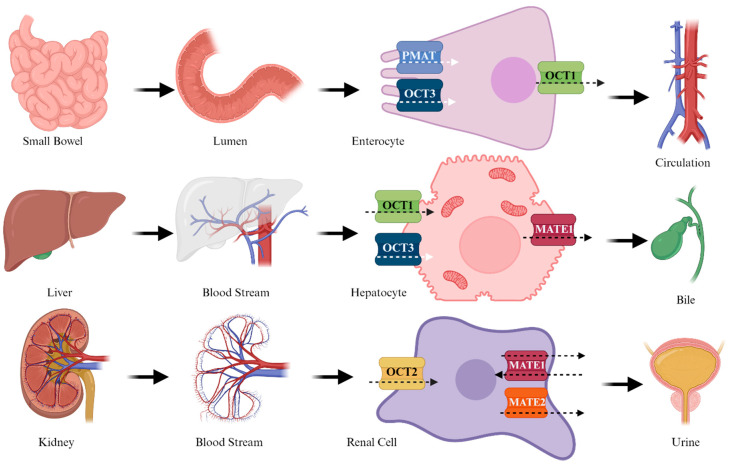
Metabolism of metformin.

Appearing as a cationic species at a physiological pH (pKa = 11.5), its potential passive diffusion across the lipid bilayer is highly improbable. Therefore, the absorption, distribution, and excretion of the molecule are facilitated by specific transporters [46,49]. At the intestinal level, absorption is facilitated by the plasma membrane monoamine transporter (PMAT) and organic cation transporter 3 (OCT3) [50]. These transporters are situated on the brush-like surface of the enterocyte membrane. Subsequently, the drug is transported into the bloodstream through OCT1, located on the basolateral side of the enterocyte. OCT1 and OCT3 are expressed on the sinusoidal membrane of hepatocytes [51], facilitating the transport of MTF into the hepatocyte. The excretion of MTF, which affects Multidrug and Toxin Extrusion 1 (MATE1), occurs thereafter. MTF clearance primarily occurs at the renal level, where OCT2, expressed in the basal cells, mediates the entry of MTF. Subsequently, at the apical level, transporters MATE1 and MATE2 [50] complete the elimination process. Notably, polymorphisms in OCT1 are likely to modify absorption, potentially diminishing the efficacy of the molecule [52] (Figure 3).

## 4. Pharmacodynamics

The target organ of MTF is the liver Within hepatocytes, MTF exerts its hypoglycemic action by inhibiting the endogenous glucose production pathway [8]. This action is mediated through the weak inhibition of complex 1 of the electron transport chain located at the mitochondrial level [53]. Complex 1 (NADH: ubiquinone oxidoreductase or NADH dehydrogenase) is a large L-shaped enzyme which facilitates the transfer of electrons from NADH to the ubiquinone pool while concurrently transferring four protons from the matrix to the intermembrane space [54]. One arm of the enzyme is embedded in the inner mitochondrial membrane, while the other extends towards the matrix. It comprises at least 45 distinct subunits, encoded by both nuclear and mitochondrial genes [55]. The inhibition of complex 1 by MTF results in decreased ATP formation, which results in more AMP than adenosyn triphosphate (ATP), leading to an elevation in the AMP/ATP ratio. Subsequently, AMP triggers the activation of AMPK (adenosine monophosphate-activated protein kinase) via LKB1 (liver kinase B1), a key regulator of cellular metabolism [56]. LKB1 is a serine/threonine kinase and tumor suppressor whose mutations have been linked to various cancers. Specifically, LKB1 codes for serine-threonine kinase 11 which, as mentioned previously, triggers the activation of AMPK and its 13 superfamily members. This activation regulates processes such as cell cycle arrest, apoptosis, and other metabolic processes related to energy production [57]. Additionally, LKB1 serves as a suppressor of inflammatory responses, a property that research indicates arises from stimulating AMPK in macrophages, suppressing the generation of pro-inflammatory mediators and chemokines [58]. Furthermore, LKB1 might regulate endoplasmic reticulum stress and macrophage autophagy through alternative pathways [59].

AMPK is responsible for various metabolic functions, including the inhibition of gluconeogenic gene transcription by phosphorylating the CREB-binding protein [55,60]. Additionally, AMPK inhibits lipogenesis by phosphorylating and deactivating acetyl-CoA carboxylase 1 (ACC1) and acetyl-CoA carboxylase 2 (ACC2), enzymes that regulate the rate of fatty acid biosynthesis by controlling the formation of malonyl-CoA. Malonyl-CoA serves as a critical precursor for fatty acid synthesis and acts as a potent allosteric inhibitor of carnitine palmitoyl transferase 1 (CPT1), which facilitates the transport of acyl-CoA into mitochondria during the β-oxidation of fatty acids [61]. Consequently, the reduction in intracellular levels of malonyl-CoA promotes fatty acid oxidation. Further contributing to its effects, MTF suppresses the SREBP-1 factor [62], which is also involved in lipogenesis. Additionally, the action of MTF seems to rely on an LKB1-AMPK-independent mechanism, as evidenced by its ability to inhibit hepatic gluconeogenesis in mice lacking AMPK [63]. Moreover, MTF noncompetitively inhibits mitochondrial glycerol phosphate dehydrogenase (mGPD), a crucial enzyme in regulating hepatic gluconeogenesis [64]. Contrary to the traditional perspective that primarily attributes MTF’s action to the liver, growing evidence supports its potential extrahepatic effects [55], particularly in the intestinal tract [8]. MTF stimulates the release of glucagon-like peptide 1 (GLP-1) [7], thereby reducing plasma glucose levels both in the fasting and postprandial states. GLP-1 is an incretin hormone composed of 30 amino acids that inhibits glucagon secretion and promotes insulin secretion, further leading to decreased satiety and serum glucose levels [65]. It is produced in the enteroendocrine L cells and secreted in response to food intake [66]. The mechanism by which MTF induces the secretion of GLP-1 is thought to be closely tied to the incretin axis; however, there is lacking evidence to support MTF’s direct effect on L cells. Another proposed theory suggests that MTF might inhibit the apical sodium-dependent bile acid transporter (ASBT) in the ileum. This inhibition could result in elevated concentrations of bile acids in the luminal environment of the terminal ileum and colon. These excess bile acids then trigger bile acid receptors, initiating a cascade that ultimately increases circulating levels of GLP-1 [67]. Additionally, supporting evidence of gut microbiome dysbiosis in patients with type 2 diabetes mellitus, MTF appears to modify the microbiome composition, contributing to its therapeutic effects [68,69].

## 5. Metformin’s Role in Ischemia-Reperfusion Injury (IRI) in the Setting of Organ Transplantation

The transplantation community has increasingly embraced the utilization of expanded criteria donors (ECDs). Arising from a growing demand for organ transplantation and a shortage of suitable organs available, transplant centers in the late 20th and early 21st centuries developed criteria expanding the pool of donors, in the attempt to reduce waiting times and increase the availability of viable organs [70,71]. ECD encompasses donors aged 60 years or older, or donors aged 50 years or older who meet two out of the following three criteria: (1) a history of hypertension, (2) a serum creatinine level ≥1.5 mg/dL, and (3) death resulting from a stroke [72]. In the United States, a new kidney allocation system, implemented in 2014, introduced the Kidney Donor Profile Index (KDPI) as its cornerstone. The KDPI is a numeric gauge that amalgamates ten factors and clinical parameters to assess the quality of deceased donor kidneys relative to others procured. This system strategically pairs kidneys with recipients predicted to have the longest post-transplant survival rates, thereby optimizing organ utilization and curbing unnecessary discards [73]. While this framework has predominantly superseded the Standard Criteria Donor (SCD) and expanded criteria donor (ECD) classifications in the U.S., it is worth noting that many other countries continue to use these terms. Conversely, lower KDPI values correlate with enhanced donor quality and anticipated longevity, as could happen with living donors [74,75].

Both recipients of high KDPI organs and ECD organs exhibit a higher rate of graft failure and shorter survival [76,77,78]. ECDs have been known to be more vulnerable to and have higher rates of IRI, prompting the adoption of diverse organ reconditioning strategies employing ex vivo perfusion techniques [79,80]. MTF’s role at the mitochondrial level has garnered interest as a potential therapeutic intervention against IRI in the perioperative setting. Specifically, attention is shifting towards investigating the potential of MTF as a component of a perfusate. An IRI is a complex and multifaceted process inherent to transplantation procedures.

**Figure 4:**
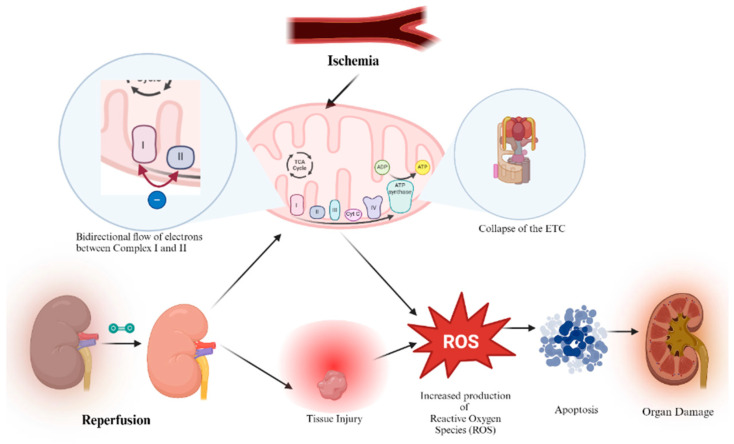
Mechanisms of ischemia-reperfusion injury.

An IRI’s occurrence stems from the interruption of blood flow during donor organ retrieval followed by subsequent reperfusion [81]. Though it is vital to reperfuse tissues via the restoration of blood flow, this step can often cause additional harm, jeopardizing the function and survival of organs and grafts. Ischemia-reperfusion injury is not limited to one organ or system, but rather can be observed in a multitude of organs and it can even trigger systemic damage to distant organs, resulting in multiple system organ failure [82]. IRI has been known to possess several mechanisms by which it can compromise endothelial and epithelial barriers and lead to said organ dysfunction and graft organ failure (Figure 4) [83]. The initial event is marked by a decline in ATP levels during the ischemic-hypoxic phase [84], leading to alterations in cellular pumps like the Na+/K+ ATPase and the Ca2+ ATPase pump. The accumulation of calcium ions subsequently disrupts mitochondrial function, resulting in the generation of reactive oxygen species (ROS), ensuing inflammation, and the opening of the mitochondrial permeability transition pore (MPT), leading to membrane potential dissipation [85].

**Figure 5:**
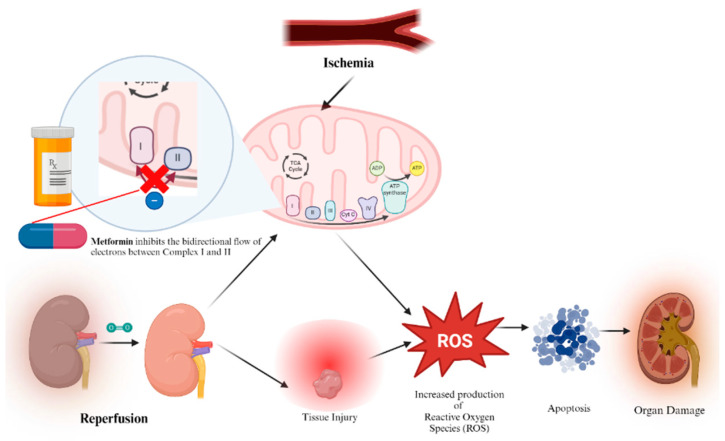
Effects of metformin on ischemia-reperfusion injury.

Superoxide anions are primarily produced at complexes 1 and 3 [86] of the electron transport chain, and it has long been recognized that electron transfer is not solely unidirectional, but can also occur in the opposite direction [87,88]. Electrons originating from complex 2 (succinate dehydrogenase) can retrogradely flow back to complex 1, resulting in the generation of ROS. It has been reported that MTF selectively inhibits this process [89] without increasing ROS formation in the forward direction. Studies have demonstrated that MTF’s inhibition of complex 1 contributes to the mitigation of myocardial ischemia-reperfusion injury (Figure 5) [90].

Additionally, AMPK, a heterodimeric kinase activated by MTF, appears to play a crucial role in attenuating IRIs [91,92,93]. Evidence suggests that the administration of MTF during the reperfusion phase can activate kinases proper to the reperfusion injury salvage kinase (RISK) pathway, which are strongly mediated by Protein kinase B (Akt) and phosphodylinositol-3-kinase (PI3K). In doing so, a protective mechanism against IRI is initiated by inhibiting the opening of the mitochondrial permeability transition pore (mPTP) during reperfusion [94].

Notably, one of the pivotal research undertaken on the subject was that conducted in 2021 by Huijink et al. in which the impact of pre- and post-conditioning with MTF on rat kidneys was studied. The rat models were subjected to 24 h of static cold storage, inducing ischemic damage. Following this, the kidneys were subjected to 90 min of normothermic machine perfusion (NMP), with or without the addition of MTF. In a separate experiment with a porcine model, the kidneys underwent NMP after 30 min of warm ischemia (WI) and 3 h of hypothermic machine perfusion (HMP) [95]. Within Huijink et al.’s model, in terms of cellular damage, rats preconditioned with MTF exhibited lower LDH values compared to a control. Additionally, postconditioning with 300 mg/L MTF resulted in the reduced necrosis and vacuolization of tubular cells. However, in porcine studies, there were no significant differences in creatinine clearance between kidneys perfused with NMP with MTF and those without the drug. Nonetheless, there was a trend towards lower creatinine levels in the metformin-treated group. While this study was well designed, several limitations should be mentioned and have been acknowledged by the authors. One limitation that hinders its clinical translational aspect is the fact that the kidneys were not transplanted. This highlights the need for future translational research, positioning the current study as an initial step towards conducting transplantation experiments. Another limitation of this particular study was the omission of measuring urinary markers of acute injury, such as kidney injury molecule 1 (KIM-1). However, the study did assess indicators of renal injury, such as lactate dehydrogenase (LDH) and aspartate aminotransferase (ASAT), which revealed modest beneficial effects from MTF treatment. Subsequent research endeavors should encompass these markers when investigating the effects of metformin IRIs.

Yu, H. et al. developed a model with male Lewis rats in which they examined the effects of MTF on lung tissue apoptosis after lung transplantation. Their results showed an attenuation in apoptosis in the metformin-treated group vs. the non-treated lung ischemia-reperfusion injury group, along with a reduction in local and systemic inflammation [96]. Ming et al.’s work further supports the protective effects of metformin on acute lung injuries in the setting of transplantation. Their work showed that metformin has the potential to yield microvascular repair via the stimulation of AMPK-α1 which alleviates pulmonary edema, decreases damage to the lung, and increases the PaO_2_/FiO_2_ ratio [97]. Several limitations in this study should be mentioned. First, brain death induction was not performed in the donor animals, thus precluding an exact replication of clinical pulmonary transplantation conditions. Secondly, technical constraints prevented the investigation of respiratory mechanics and related data. Thirdly, the study primarily remained descriptive, lacking in-depth in vitro experimental validation and molecular mechanism exploration. Despite these limitations, the findings presented in this body of work served as an important foundation for future experimental studies.

Westerkamp et al.’s work investigated the effects of MTF preconditioning on the incidence of hepatobiliary injury and hepatobiliary function in rat liver transplantation models. Their work showed that preconditioning with MTF lowers this incidence and improves hepatobiliary function post-transplantation [98]. While their findings are promising, the authors acknowledge that the experimental design needs further testing in animal models with more severe hepatobiliary injuries to further evaluate the effectiveness of MTF in these specific situations. Again, this study is a great foundation for further research investigating the role of MTF in the setting of IRIs and organ transplantation.

## 6. Conclusions

Overall, there is a growing body of evidence supporting the possibility of using MTF in the transplantation setting, due to its potential to recondition and protect against IRI, by attenuating free radical damage, activating AMP-activated protein kinase to preserve cellular energy and promote repair, as well as directly reducing inflammation and enhancing microcirculation. Its role in the activation of the RISK pathways seems to be the most notorious feature of all and one which merits further exploration. However, its actual future impact on organ quality and viability still remains unclear and is difficult to predict. The literature is still lacking and while strong foundational experimental studies have been attempted, these have not been without limitations. Further studies are necessary to better elucidate the effects of this pleiotropic drug, as well as studies to further translate current findings.

MTF remains a mainstay of diabetes management. It is a cost-effective, accessible, and overall well-tolerated drug. Should additional evidence emerge to endorse its utilization in transplantation, whether as a constituent of a perfusate or as part of preoperative protocols preceding the organ implant, the ramifications could be immense, yielding a decrease in organ transplantation failures, decreasing morbidity and mortality and improving patient outcomes globally with the implementation of a low-cost and already widely used drug.

With an increasing trend in the number of transplants being performed worldwide, demonstrated by the 46,632 organ transplants performed solely in the US in 2023 and the current high rates of IRI in transplantation, we must continue to explore methods to prevent organ graft failure and enhance the success rate of organ transplantation and the mitigation of IRIs.

Future directions related to MTF in organ transplantation should include additional studies evaluating MTF preconditioning in animal models that can be replicated, evaluate more severe forms of ischemia/pathology, and take into account additional markers of injury for a more comprehensive understanding of the role of MTF in the mitigation of IRI. Translational studies must also be conducted, followed by the evaluation of various possible perfusates incorporating MTF as a main component to further design protocols with specific dosing strategies and guidelines for implementation in the clinical setting.

## Abbreviations

ACC	acetyl-CoA carboxylase
AMP	adenosine monophosphate
AMPK	adenosine monophosphate-activated protein kinase
ASBT	sodium-dependent bile acid transporter
ATP	adenosine triphosphate
CPT	carnitine palmitoyl transferase
ECD	extended criteria donor
FDA	Food and Drug Administration
GI	gastrointestinal
GLP	glucagon-like peptide
HbA1c	glycated hemoglobin
HMP	hypothermic machine perfusion
IRI	ischemia-reperfusion injury
LKB1	liver kinase B1
KDPI	Kidney Donor Profile Index
mGPD	glycerol phosphate dehydrogenase
MATE	multidrug and toxin extrusion
MPT	mitochondrial permeability transition pore
MTF	metformin
NADH	nicotinamide adenine dinucleotide + hydrogen
NMP	normothermic machine perfusion
mTORC1	mechanistic target of rapamycin complex 1
OCT	organic cation transporter
PMAT	plasma membrane monoamine transporter
RISK	Reperfusion injury salvage kinase
ROS	reactive oxygen species
SCD	Standard Criteria Donor
T2DM	type 2 diabetes mellitus
WHO	World Health Organization
